# junctionCounts: comprehensive alternative splicing analysis and prediction of isoform-level impacts to the coding sequence

**DOI:** 10.1093/nargab/lqae093

**Published:** 2024-08-09

**Authors:** Alexander J Ritter, Andrew Wallace, Neda Ronaghi, Jeremy R Sanford

**Affiliations:** Department of Biomolecular Engineering, University of California Santa Cruz, Santa Cruz, CA 95064, USA; Department of Molecular, Cell and Developmental Biology, University of California Santa Cruz, Santa Cruz, CA 95064, USA; Department of Molecular, Cell and Developmental Biology, University of California Santa Cruz, Santa Cruz, CA 95064, USA; Department of Molecular, Cell and Developmental Biology, University of California Santa Cruz, Santa Cruz, CA 95064, USA

## Abstract

Alternative splicing (AS) is emerging as an important regulatory process for complex biological processes. Transcriptomic studies therefore commonly involve the identification and quantification of alternative processing events, but the need for predicting the functional consequences of changes to the relative inclusion of alternative events remains largely unaddressed. Many tools exist for the former task, albeit each constrained to its own event type definitions. Few tools exist for the latter task; each with significant limitations. To address these issues we developed junctionCounts, which captures both simple and complex pairwise AS events and quantifies them with straightforward exon-exon and exon-intron junction reads in RNA-seq data, performing competitively among similar tools in terms of sensitivity, false discovery rate and quantification accuracy. Its partner utility, cdsInsertion, identifies transcript coding sequence (CDS) information via *in silico* translation from annotated start codons, including the presence of premature termination codons. Finally, findSwitchEvents connects AS events with CDS information to predict the impact of individual events to the isoform-level CDS. We used junctionCounts to characterize splicing dynamics and NMD regulation during neuronal differentiation across four primates, demonstrating junctionCounts’ capacity to robustly characterize AS in a variety of organisms and to predict its effect on mRNA isoform fate.

## Graphical Abstract

**Graphical Abstract:**
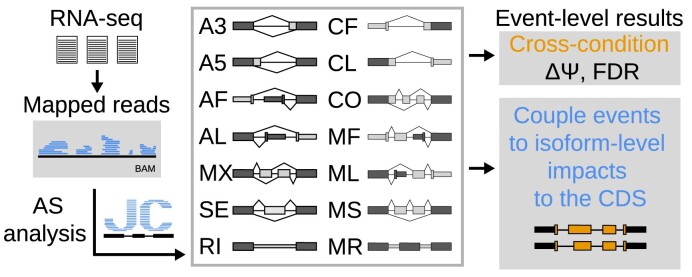

## Introduction

Alternative splicing (AS) generates a diverse array of mRNA isoforms from a single locus. The consequences of this process can have manifold effects on gene expression by altering mRNA half-life (1), intracellular localization (2), translation efficiency (3), and most obviously, by producing different protein isoforms (4). AS plays critical roles in a variety of biological processes including disease pathology (5), cancer (6) and cellular differentiation (7). In cellular homeostasis, AS is tightly regulated to control the precise expression of diverse mRNA isoforms, allowing cells to adapt to changing conditions and to achieve different states of activation in immune cells, for example (8). Regulatory elements, including *cis*-acting splicing enhancers and silencers, as well as *trans*-acting splicing factors, coordinate the inclusion or exclusion of alternative exons or splice sites during transcription (9).

Dysregulation of AS can contribute to the production of aberrant protein isoforms, impacting critical cellular functions. In cancer, this can result in the generation of oncogenic isoforms, altered signaling pathways and evasion of regulatory mechanisms (10). Often, mutations in splicing factors and in *cis*-elements underlie oncogenic AS dysregulation (11). During cellular differentiation from pluripotent stem cells, AS orchestrates the precise control of gene expression, directing the development of specialized cell types with distinct functions (12). This process is intricately involved in shaping the cellular landscape, driving fate decisions, and maintaining tissue homeostasis (13). The important impact of AS in disease and cellular differentiation underscores its significance as a regulatory force in biological processes and highlights its potential as a therapeutic target in pathological conditions.

It is thus important, in any eukaryotic cellular context, to understand gene expression at the isoform level. The complex nature of AS, however, creates numerous obstacles to its accurate study. mRNA isoforms can, and have canonically been, characterized in terms of binary events that either include or exclude an alternative feature (exon, intron or splice site). This mode of characterization faces the challenge of differentiating between complex and overlapping event features, and also relies on comprehensively annotated gene structures. The latter problem is highlighted by the lack of records for transcripts clearly supported by mapped reads in available references. To address this, reference-guided or *de novo* transcriptome assembly has become a widely used step in RNA-seq analysis. In this process, transcript structure is predicted from the data with or without the use of a reference gene annotation as a template (14). This process allows analysts to consider potential novel transcripts and novel alternative event features that may be important to the biological phenomena under study.

Upon establishing comprehensive gene models, it subsequently becomes important to understand how AS configures the coding and noncoding regions of resultant mRNA isoforms. Unfortunately, common tools for transcriptome assembly (15,16) are unable to provide information on the presence and nature of open reading frames (ORFs) that may be contained within predicted novel transcripts. One example of an available tool, Transdecoder (17), somewhat addresses this limitation—however, it was developed for use with Trinity (17) which is intended for completely *de novo* transcriptome assembly in the absence of a reference genome assembly or any annotations. Consequently, Transdecoder performs *de novo* ORF prediction with intent towards identifying all ORFs that could convincingly give rise to proteins. In most well-annotated genomes, however, existing ORF annotations are available for the majority of genes in which new transcripts might be identified. In these cases a potentially more reliable approach is to examine novel coding sequences that begin with high-confidence annotated start codons. This prediction approach is useful not only because it informs on potential novel peptides, but also because it has the ability to identify the presence of premature termination codons (PTCs) or high-confidence start codons that lack an in-frame downstream stop codon. In these latter cases, translation is expected to result in surveillance of host transcripts via nonsense-mediated decay (NMD) and non-stop decay (NSD) respectively.

NMD exemplifies an important potential outcome of AS. This translation-dependent surveillance mechanism identifies transcripts containing PTCs ≥ 55 nt upstream of a splice junction and triggers its degradation and translational suppression (18). PTCs can be introduced through AS by inclusion of PTC-containing exons (poison exons), through splicing events that shift the reading frame of the message and by splicing events occurring within the 3′ untranslated region (3′UTR) (19). Other outcomes that can dramatically affect the fate of mRNAs may involve coding-to-noncoding switches, long-to-short UTR switches, or the inclusion of rare codons. It is thus important to profile mRNA coding features, and to connect binary AS events to their potential impacts to mRNA fate and function. The functional impacts of AS, however, remain difficult to predict.

To address this problem we developed junctionCounts comprising: junctionCounts event identification and quantification modules, cdsInsertion and findSwitchEvents. junctionCounts identifies and quantifies a diverse array of AS events. cdsInsertion translates provided transcripts *in silico* from user-provided overlapping start codons and determines resulting transcript characteristics such as UTR lengths, putative primary structure, the presence of PTCs, PTC distances from downstream splice sites, and more. Its partner utility, findSwitchEvents, bridges the gap between isoform- and event-level analysis, allowing one to take advantage of the potential superior quantification accuracy and regulatory interpretation of event-level analysis while still leveraging information that can only be derived from full-length transcripts (20). In this study, we present junctionCounts as a powerful and flexible tool for studying AS in a variety of cellular contexts, and we demonstrate its utility in not only identifying significantly regulated splicing events, but also inferring their functional outcomes.

## Materials and methods

### Alternative event definition and classification in junctionCounts

Alternative events are defined as instances in which pairs of: (a) identical upstream 5′- and identical downstream 3′-exon boundaries, (b) non-identical upstream 5′-transcript termini and identical downstream 3′-exon boundaries or (c) identical 5′-exon boundaries and non-identical 3′-transcript termini are separated by any two combinations of distinct exon coordinates. Cases in which the aforementioned pairs are separated by more than two sets of distinct exon coordinates result in distinct alternative events for all pairwise combinations of those sets. Cases in which two or more events share the same splicing structure result in a single representative event in which the most proximal outer exon boundaries are used. This approach to event identification encompasses standard alternative event types and further identifies non-standard events of complex exon structure.

**Figure 1.**
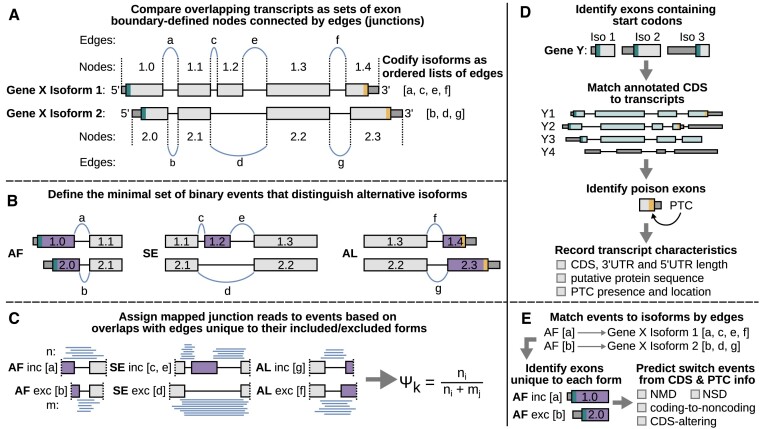
Overview of junctionCounts and partner utilities: cdsInsertion and findSwitchEvents. (**A**) junctionCounts takes a transcriptome annotation and makes pairwise comparisons of overlapping transcripts within genes, handling them as nodes (exons) connected by edges (junctions). (**B**) This iterative process identifies and classifies the minimal set of binary events that differentiates alternative isoforms. (**C**) junctionCounts then assigns mapped junction reads to the unique junctions that define the included and excluded forms of events and quantifies percent spliced in (PSI) values based on the ratio of included form and total junction reads mapping to the event. (**D**) cdsInsertion identifies exons overlapping annotated start codons, matches annotated coding sequences (CDS) to transcripts, identifies exons containing PTCs (poison exons), and finally records isoform-level information about the CDS. (**E**) Lastly, findSwitchEvents matches the included and excluded forms of events to isoforms, distinguishes the unique exons of each form, and predicts the impact of an event's inclusion/exclusion on the CDS. These predictions specify events whose inclusion/exclusion may confer: NMD, NSD, a coding-to-noncoding switch, or simply an alteration of the CDS relative to its opposite form.

junctionCounts begins by identifying alternative events solely from a user-provided transcriptome annotation, in contrast to many other tools that also require BAM files, via its event identification module: infer_pairwise_events.py. This approach allows users to generate a single event catalog for the latest GENCODE transcriptome annotation (21), for example, and to use it across multiple datasets involving samples from a common species. If detection of novel splice sites and consideration of unannotated exons is desired, users can assemble a transcriptome *de novo* from the RNA-seq data under study with a tool like StringTie (16) and provide it as input to the event identification module. junctionCounts begins inferring pairwise events by generating a dictionary of annotated transcripts containing chromosome and strand information, a sorted list of exon coordinates and a sorted list of accompanying junction coordinates which are simply the inner coordinates of any pair of exons. It then filters the transcript dictionary based on user-defined cutoffs with the following defaults: exon length within 3 nt–35 kb and intron length within 20 nt–1 Mb. Then, it codifies transcripts as nodes based on exon boundaries, connected by edges based on junction coordinates (Figure 1A). After establishing these simplified transcript structures, it makes pairwise comparisons of overlapping transcripts and iteratively defines the unique nodes that differentiate them; ultimately arriving at the minimal set of binary events that distinguish alternative isoforms in a gene (Figure 1B). During this process, events are also classified based on the nature of component features. The majority of event types in junctionCounts correspond to types explicitly defined elsewhere (22), but it also adopts previous usage of the term ’complex’ (23,24) to refer to non-standard event types, distinguishing between internal (CO), 5′-terminal (CF) and 3′-terminal (CL) contexts. Below are descriptions of the criteria that define each event type:

**A3**—*alternative 3′-splice site*: an event in which an upstream exon and the 3′-boundary of the downstream exon are common to both isoforms, but the 3′-splice site is distinct. The form containing the most upstream of the alternative 3′-splice sites is the included form.
**A5**—*alternative 5′-splice site*: an event in which a downstream exon and the 5′-boundary of the upstream exon are common to both isoforms, but the 5′-splice site is distinct. The form containing the most downstream of the alternative 5′-splice sites is the included form.
**AF**—*alternative first exon*: an event in which each isoform has its own distinct 5′-terminal exon; each with a distinct 5′-terminus and 3′-splice site. The exon immediately downstream of the terminal exon is common to both isoforms. The form with the most upstream 5′-terminus is the included form.
**AL**—*alternative last exon*: an event in which each isoform has its own distinct 3′-terminal exon (i.e. each with a distinct 3′-terminus and 5′-splice site). The exon immediately upstream of the terminal exons is common to both isoforms. The form with the most downstream 3′-terminus is the included form.
**MX**—*mutually exclusive exons*: an event in which a pair of 3′- and 5′-splice sites are separated by a distinct exon in each isoform. The only requirement for the two exons being distinct is that they do not share either splice site. It is possible for the alternative exons in an MX event to partially and completely overlap, provided their boundaries do not coincide. The form in which the alternative exon's 3′-splice site is the most upstream of the mutually exclusive exons is the included form.
**RI**—*retained intron*: an event in which a pair of adjacent exons are spliced together in one isoform, but joined together in another by retention of the intron separating them. The form with the retained intron is the included form.
**SE**—*skipped exon*: an event in which a pair of 3′- and 5′-splice sites are separated by a single exon (the skipped exon) in one isoform and spliced directly together in another. The form containing the intermediate exon is the included form.
**MF**—*multiple alternative first exons*: an event in which each isoform has its own distinct 5*′*-terminal set of one or more exons upstream of a single shared exon. This event type is distinguished from AF in that either isoform must contain more than one unique exon. The form with the most upstream 5′-terminus is the included form.
**ML**—*multiple alternative last exons*: an event in which each isoform has its own distinct 3′-terminal set of one or more exons downstream of a single shared exon. This event type is distinguished from AL in that either isoform must contain more than one unique exon. The form with the most downstream 3′-terminus is the included form.
**MR**—*multiple retained intron*: an event in which a set of three or more exons are spliced together in one isoform, but connected in the other by two or more consecutive retained introns. The form with the retained introns is the included form.
**MS**—*multiple skipped exons*: an event in which a pair of 3′- and 5′-splice sites are separated by multiple exons in one isoform, and spliced directly together in another. The form containing the intermediate exons is the included form.
CO*—*complex internal*: this is a general category for events that do not meet any of the above criteria and do not involve transcript termini. The isoform with the longest spliced length is the included form.
CF*—*complex 5′-terminal*: this is a general category for events that do not meet any of the above criteria and involve alternative 5′ transcript termini. The form with the most upstream 5′-terminus is the included form.
CL*—*complex 3′-terminal*: this is a general category for events that do not meet any of the above criteria and involve alternative 3′ transcript termini. The form with the most downstream 3′-terminus is the included form.
*Complex event types capture less straightforward cases of binary splicing events that can involve combinations of multiple alternative features (terminal and internal exons and/or splice sites).

After generating the initial event dictionary, junctionCounts writes BED files for introns and exons separately and uses bedtools intersect (25) with the arguments -wa -wb -s -f 1.00 to identify introns that completely overlap an exon. These introns are then considered putative RI events. Finally, junctionCounts organizes events by edges (junctions) that are unique to their included and excluded forms, collapses any redundant events that have identical included and excluded form edges, and filters them for events that can be quantified by exon-exon junction reads alone (or by exon-intron junction reads in the case of RI/MR events).

### Alternative event quantification in junctionCounts

junctionCounts employs a junction read-centric approach to alternative event quantification. First, the event quantification module, junctionCounts.py, generates a nested containment list (26) of all event junction coordinates. Then, for each read or read pair, junctionCounts considers matches between splice junctions identified in the alignment and event splice junctions, as well as overlaps between contiguous mapped read sequence and informative exon-intron junctions (Figure 1C). Informative exon-intron junctions are those that are overlapped by an exon in the alternative isoform. Reads overlapping such an exon-intron junction are considered consistent with the alternative isoform. Key examples of this occur in the excluded isoform of RI events, which are overlapped by the exon of the included form. After establishing the event isoforms with which a read is consistent, junctionCounts attempts to disambiguate the read assignment using exon-exon and exon-intron junctions that are unique to specific isoforms, when possible. With this approach, junctionCounts goes beyond simple junction-by-junction read counting. Both the event and the informative exon-intron junction definition prohibit scenarios in which reads are assigned to both isoforms of the same event. With read-to-event isoform consistencies established, read counts are tallied for each exon-exon and informative exon-intron junction for each isoform of each event. A percent spliced in (PSI or Ψ) value is calculated for all pairwise combinations of included and excluded junction counts, yielding the ratio between an included form's junction counts and the sum of the included *and* excluded form's junction counts:


(1) \begin{equation*}{{\mathrm{\Psi }}}_{\mathrm{k}} = \frac{{{{\mathrm{n}}}_{\mathrm{i}}}}{{{{\mathrm{n}}}_{\mathrm{i}}\ + \ {{\mathrm{m}}}_{\mathrm{j}}}}\end{equation*}

where *n_i_* is the number of reads assigned to the included form junction *i* and *m_j_* is the number of reads assigned to the excluded form junction *j*. A set of PSI values is established for each sample. Additionally, junctionCounts calculates the minimum (Ψ_min_), maximum (Ψ_max_) and span PSI (Ψ_span_):


(2) \begin{equation*}{{\mathrm{\Psi }}}_{{\mathrm{min}}} = {\mathrm{min}}({{\mathrm{\Psi }}}_{\mathrm{k}})\end{equation*}

(3) \begin{equation*}{{\mathrm{\Psi }}}_{{\mathrm{max}}} = {\mathrm{max}}({{\mathrm{\Psi }}}_{\mathrm{k}})\end{equation*}

(4) \begin{equation*}{{\mathrm{\Psi }}}_{{\mathrm{span}}} = {{\mathrm{\Psi }}}_{{\mathrm{max}}} - {{\mathrm{\Psi }}}_{{\mathrm{min}}}\end{equation*}

The Ψ_span_ serves as a rough measure of within-sample uncertainty. junctionCounts reports these values as well as the included and excluded junction read counts (*n* and *m* in Equation 1) for each event. As an optional method of assessing within-sample uncertainty, junctionCounts offers bootstrap quantification, which repeats a user-specified number of rounds of bootstrap read selection and re-quantification. For each bootstrap round, junctionCounts reports all measurements, in addition to the initial non-resampled quantification.

Statistical testing of events between conditions in experiments with at least two replicates per condition is done with the condition comparison module, DEXSeq_comparison.R, which employs DEXSeq (27). First, it produces a DEXSeq-compatible GFF file that defines the included and excluded form of each event as pairs of ‘exonic parts’. It subsequently writes included and excluded form junction counts per sample as separate count files. DEXSeq then normalizes included and excluded junction counts as per its documentation (27), and estimates included and excluded form junction count dispersions using a Cox–Reid adjusted profile likelihood estimation followed by fitting of a dispersion-mean relation to the individual dispersion values and shrinkage of per-form estimates toward fitted values. Next, DEXSeq compares the deviances of the included and excluded form junction counts in each event across conditions using a *χ*^2^-distribution to produce *P*-values for each form. Event-level *Q*-values are then calculated from the *P*-values with the Benjamini–Hochberg procedure. Finally, events are filtered based on user-defined cutoffs with the following defaults: ≥0.1 mean PSI in at least one condition, ≥15 total mean junction counts across all replicates and ≤0.03 span PSI for RI/MR events. The final results provide event coordinates, quantification results, and classification of events as significant based on user-defined cutoffs with the following defaults: |dPSI| ≥ 0.1 and *Q*-value ≤0.05.

### Description and validation of partner utilities: cdsInsertion and findSwitchEvents, which couple alternative splicing events to isoform-level impacts to the CDS

cdsInsertion translates transcripts *in silico* from user-provided overlapping start codons and determines resulting transcript characteristics including: UTR lengths, putative protein sequences, the presence of PTCs, PTC distances from downstream splice sites and more (Figure 1D). For a given codon and transcript, cdsInsertion first checks whether the start codon's first position overlaps the genomic coordinates of a transcript's exons. If it does, the start codon sequence is checked in the spliced transcript's sequence. Currently, only AUG initiation is supported. If the start codon sequence is AUG, cdsInsertion translates the spliced transcript *in silico* by looking for an in-frame downstream stop codon, which can be: UAA, UGA or UAG. If a downstream stop codon is found, the resulting CDS is recorded along with associated information such as CDS length, coding sequence, PTC presence and PTC distance. If more than one CDS is found within the transcript, additional CDS features are associated to the given transcript as distinct CDS features. If no in-frame downstream stop codon is found, the transcript is recorded as a possible NSD substrate. cdsInsertion outputs a table with summary information about each transcript and a separate GTF file for potential non-PTC, PTC, and non-stop transcript-CDS combinations. The GTF file contains separate transcript records for every CDS-transcript combination. Optionally, cdsInsertion can additionally output bigGenePred files which enable codon visualization on the UCSC Genome Browser. cdsInsertion further outputs a pickled Python dictionary containing all of the aforementioned information associated with each transcript.

**Figure 2.**
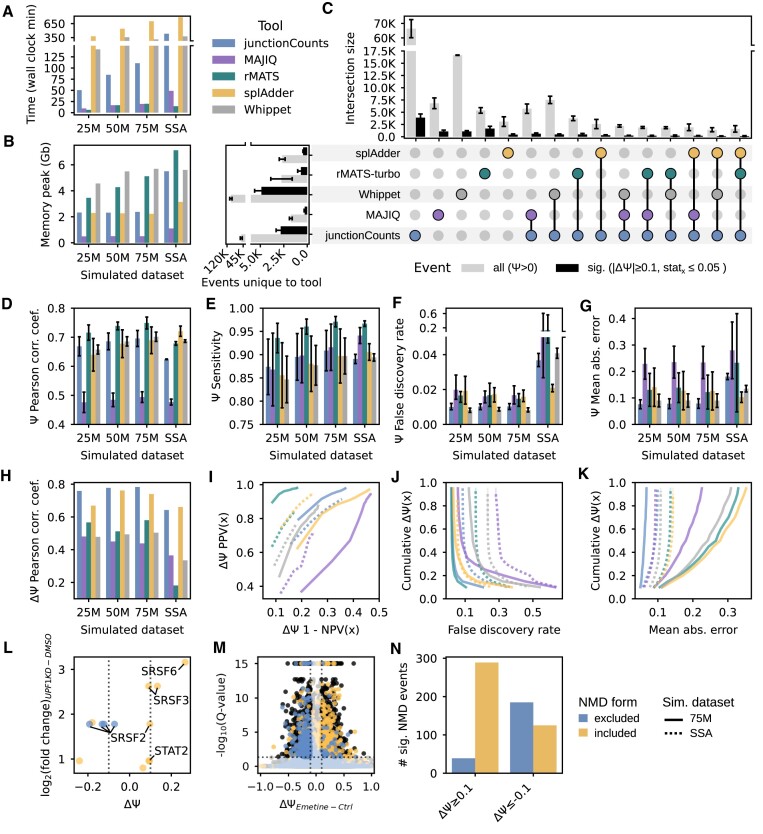
Benchmarking experiment to evaluate junctionCounts performance relative to similar tools. Four simulated datasets were generated: 25M, 50M and 75M refer to the library depths of samples simulated from mouse cerebellum and liver RNA-seq in triplicate for each cell type. SSA refers to samples simulated from human cells treated with spliceostatin A or DMSO in triplicate for each condition, at 50M reads per library. dPSI measurements were made across cell types in the mouse data and across conditions in the human data respectively. (**A**) Measurements of elapsed wall clock time upon running full tool pipelines to completion on all 6 samples of each simulated dataset. (**B**) Peak memory consumption at any point of each tool's full pipeline. (**C**) Upset plot showing the intersection of each tool's alternative events with junctionCounts-defined ground truth events in the 25M, 50M and 75M datasets. Gray bars represent all events detected by a given tool or set of tools which had PSI > 0 in at least one condition. Black bars represent events determined to be differentially spliced across conditions by a given tool or set of tools (|dPSI| ≥ 0.1 and the tool's associated statistic meeting a probability of 0.95 or a FDR/*P*-value of 0.05 for the event). The horizontal barplot on the left shows the number of events that didn’t overlap between each tool and junctionCounts – or in the case of junctionCounts itself, the number of events that were not reproduced by any other tool. (**D**) Pearson correlation coefficient of measured PSI values for each sample compared with its cognate ground truth (6 total replicates and comparisons per dataset). (**E**) Sensitivity and (**F**) false discovery rate of event detection. (**G**) Mean absolute error of PSI measurements. Error bars for (D, E and G) depict standard deviation, while those for (**F**) show the full range of observations. (**H**) Pearson correlation coefficient of measured dPSI values derived from each tool's respective condition comparison steps with accompanying statistical filtering (|dPSI| ≥ 0.1 and *Q*-value/*P*-value ≤ 0.05 or probability ≥ 0.95). (**I**) Predictive receiver operating characteristic (PROC) showing positive predictive value (PPV) and negative predictive value (NPV) of significant dPSI calls at cumulative ground truth dPSI thresholds. (**J**) False discovery rate of dPSI calls at cumulative ground truth dPSI thresholds. (**K**) Mean absolute error of dPSI measurements at cumulative ground truth dPSI thresholds. (I–K) Only the 75M and SSA datasets (solid and dotted lines respectively) are shown because the 25M and 50M datasets had nearly identical curves to 75M. (**L**) RT-PCR log_2_(fold change) of NMD targets in UPF1-KD HEK-293 cells vs. DMSO compared with dPSI measurements of junctionCounts-predicted NMD events within those targets. (**M**) Volcano plot of junctionCounts-predicted NMD events in emetine-treated HEK-293 T cells versus DMSO. (**N**) The number of significant predicted NMD events stratified by dPSI directionality upon emetine treatment in HEK-293 T cells. Events in (L–N) are categorized as included or excluded NMD form, meaning that the included form or the excluded form, respectively, is predicted to confer NMD to the resulting transcript. Performance metrics in (E–G and I–K) are described in detail in the Materials and Methods.

Its partner utility, findSwitchEvents, takes an IOE file; a format originally introduced by SUPPA (28), which is generated by junctionCounts to associate events with transcripts, and the pickled Python dictionary containing transcript CDS information and associated details from cdsInsertion. With this information, it evaluates whether isoforms with a specific property (NMD, NSD or unique CDS) are exclusive to one form of an alternative event (Figure 1E). Switch events are AS events that meet this condition, meaning that one (either the included or excluded) form is coupled with a switch to PTC-containing, non-stop or noncoding isoforms within a gene. We evaluated these tools on two published datasets. First, we procured RT-PCR data for well-characterized NMD targets and accompanying RNA-seq data from HEK-293 cells upon UPF1 knockdown versus non-targeting siRNA (29,30). We then ran junctionCounts and its partner utilities with default settings on the RNA-seq data to predict NMD switch events within the NMD targets verified with RT-PCR in the original study. Out of the 13 predicted NMD switch events associated with the NMD targets, 11 had the expected dPSI directionality (Figure 2L). Additionally, we used the same approach on a dataset in which treatment with emetine, a translation elongation inhibitor, was reported to increase the abundance of NMD substrates in HEK-293 T cells (31). Indeed, cdsInsertion and findSwitchEvents identified 636 potential NMD switch events with significant changes in splicing (|dPSI| ≥ 0.1 and *Q*-value ≤ 0.05) upon emetine treatment, out of which 472 (74.2%) exhibited the expected dPSI directionality (Figure 2M, N).

### Benchmarking performance across five AS analysis tools

junctionCounts was evaluated on its performance relative to four established splicing analysis tools: MAJIQ v2.5.6.dev1 + g8423f68b (32), rMATS-turbo v4.3.0 (33), splAdder v3.0.4 (34) and Whippet v1.6.2 (20). We generated four paired-end simulated datasets from real RNA-seq data using polyester v1.36.0 (35) with the arguments: read.length = 100, fragment.length.min = 100, fragment.length.max = 500, fragment.length.mean = 180, fragment.length.sd = 40 and simulated.sequencing.error = TRUE. Three of the simulated datasets: 25M, 50M and 75M, were used to evaluate performance at different library depths (25, 50 and 75 million reads per library respectively). These datasets were modeled on mouse cerebellum and liver RNA-seq data in triplicate for each cell type from Vaquero-Garcia et al. (2016) (32), who replicated the experiments in Zhang *et al.* (36). The fourth simulated dataset was modeled on human RNA-seq data from HeLa cells treated with either spliceostatin A or DMSO (29) at 50 million reads per library with triplicates for each condition. This dataset introduced a larger pool of potential AS events to test compared to the murine simulated datasets, and importantly increased the total number of MR events tested.

To generate these simulated datasets, we first downloaded the FASTQ files for the aforementioned mouse and human RNA-seq data (29,32) from their respective data repositories and verified their quality with FASTQC v.0.12.1. Next, we mapped them to their respective genomes and transcriptomes (GRCm38.p6 with the GENCODE vM20 primary assembly annotation for mouse data and GRCh38 with the GENCODE v41 primary assembly annotation for human data) (21) with STAR v2.7.8a (37). Then, we quantified transcript-level expression with Salmon v.1.10.2 (38). The transcript-level quantification results were used as input for polyester to simulate RNA-seq libraries reflecting the exact transcripts per million (TPM) specified for each sample in each simulated dataset – thus producing datasets with known ground truth transcript expression values. Ground truth PSI and dPSI values for junctionCounts-defined AS events were calculated from the TPM values with a custom Python script that leverages the event-transcript associations in the junctionCounts IOE file using the following equation:


(5) \begin{equation*}{\mathrm{\Psi }} = \frac{{\sum\nolimits_{{\mathrm{i}} = 1}^{\mathrm{n}} {{\mathrm{TP}}{{\mathrm{M}}}_{\mathrm{i}}} }}{{\sum\nolimits_{{\mathrm{i}} = 1}^{\mathrm{n}} {{\mathrm{TP}}{{\mathrm{M}}}_{\mathrm{i}}} + \sum\nolimits_{{\mathrm{j}} = 1}^{\mathrm{m}} {{\mathrm{TP}}{{\mathrm{M}}}_{\mathrm{j}}} }}\end{equation*}

where transcripts *i* in the numerator are those consistent with the included form of the event, and transcripts *j* in the denominator are those consistent with the excluded form of the event. We then mapped the simulated data to the appropriate genomes with STAR (37) and evaluated each tool's performance on the resulting BAMs (and FASTQ files in the case of Whippet). All tools were run on a System76 Lemur Pro laptop with an Intel® Core™ Ultra 5 125U @4.3GHz processor, 14 total cores and 40GB RAM. The total time elapsed for all steps of each tool's pipeline on all 6 samples of each dataset and the memory peak among all steps of each tool's pipeline were measured with the default linux package ‘time’ (http://man7.org/linux/man-pages/man1/time.1.html) with the ‘-v’ flag. Below are the run parameters for each tool with specific arguments and flags noted, excluding arguments related to user-specific input/output files and directories. The following variables refer to either the mouse or human versions of genome sequences and annotations: ‘$GTF’ (GENCODE vM20/GENCODE v41 primary assembly annotation GTF) ‘$GFF3’ (GENCODE vM20/GENCODE v41 primary assembly annotation GFF3) and ‘$FASTA’ (GRCm38.p6/GRCh38 genome sequence FASTA).

junctionCounts was run with default settings:

1. Event identification step: infer_pairwise_events.py –transcript_gtf $GTF
2. Event quantification step: junctionCounts.py
3. Condition comparison step: DEXSeq_comparison.R

MAJIQ:

1. Event identification step: majiq build $GFF3 –minreads 5
2. Event quantification step: majiq psi
3. Condition comparison step: majiq deltapsi
4. Produce output TSV file: voila tsv –changing-between-group-dpsi 0.1 –threshold 0.1
5. Modulize Voila files: voila modulize –show-all –changing-between-group-dpsi 0.1

rMATS-turbo:

1. All steps: rmats.py –gtf $GTF -t paired –libType fr-secondstrand –readLength 100 –variable-read-length –nthread 4

splAdder:

1. Event identification and quantification step: spladder build -a $GTF –remove-se –validate-sg-count 3 –confidence 2
2. Condition comparison step: spladder test –confidence 2

Whippet:

1. Preparation step (not included as part of Whippet's time/memory evaluation because it's not a required step): generate a merged, deduplicated and indexed BAM file from all the sample BAMs as input with samtools merge, sort, rmdup and index commands.
2. Event identification step: julia whippet-index.jl –fasta $FASTA –bam merged.sort.rmdup.bam –gtf $GTF –suppress-low-tsl –bam-min-reads 5
3. Event quantification step: julia whippet-quant.jl –biascorrect
4. Condition comparison step: julia whippet-delta.jl

The next step to evaluate performance on the simulated datasets was to identify events defined by each tool that approximately reproduce the junctionCounts-defined events for which we have ground truth PSI values. Each tool defines events with their own unique and valid approach, leading to many events with slightly different exon/splice site/intron node edges across tools. Therefore, each tool was evaluated on its individual subset of events that matched junctionCounts-defined events in the simulated datasets with ≥95% overlap at each participating alternative feature. Performance at the event detection and quantification level (PSI) and at the event change level (dPSI) were then assessed in terms of the following metrics with specific adjustments to maximize fairness:


(6) \begin{equation*}{\mathrm{TPR}} = \frac{{{\mathrm{TP}}}}{{{\mathrm{TP}}\ + {\mathrm{FN}}}}\end{equation*}

(7) \begin{equation*}{\mathrm{FDR}} = \frac{{{\mathrm{FP}}}}{{{\mathrm{FP}} + {\mathrm{TP}}}}\end{equation*}

(8) \begin{equation*}{\mathrm{MAE}} = \frac{{\sum \left| {{\mathrm{GT}} - {\mathrm{\ OB}}} \right|}}{{\mathrm{n}}}\end{equation*}

(9) \begin{equation*}{\mathrm{PPV}} = \frac{{{\mathrm{TP}}}}{{{\mathrm{TP}}\ + \ {\mathrm{FP}}}}\end{equation*}

(10) \begin{equation*}{\mathrm{NPV}} = \frac{{{\mathrm{TN}}}}{{{\mathrm{TN}}\ + {\mathrm{FN}}}}\end{equation*}

For event detection and quantification (PSI) metrics, we measured: sensitivity (TPR), false discovery rate (FDR) and mean absolute error (MAE). True positives (TP) were defined as events for which the ground truth (GT) and a given tool's measured/observed PSI values (OB) were both > 0. False negatives (FN) were defined as events with GT PSI > 0 and OB PSI = 0. False positives (FP) were defined as events with GT PSI < 0.05 and OB PSI ≥ 0.05. We chose this definition of FP because each tool commonly reported miniscule OB PSI values <0.05 for events with GT PSI = 0, which would in most normal use cases be excluded or filtered in subsequent analyses unless they had more substantial (typically ≥ 0.1) PSI values in another condition. TPR and FDR were calculated with the described definitions of TP, FP and FN. MAE was calculated by summing the absolute differences between GT and OB PSI values for TP events and dividing it by the number of observations, *n*.

For event change (dPSI) metrics, we measured: positive predictive value (PPV), negative predictive value (NPV), FDR and MAE. Each metric was measured at cumulative |dPSI| thresholds starting at 0.1 and increasing stepwise by 0.05 to 1.0, such that each measurement considers the subset of events with |*GT* dPSI| both at and below the given threshold. Each tool was evaluated based on its accuracy of significant/insignificant calls and quantification of event changes. Ground truth events with |GT dPSI| ≥ 0.1 were considered significant. Therefore, each tool's condition comparison step was given the appropriate argument specifying a dPSI threshold of 0.1 to be considered statistically significant. Significant events were defined for each tool as those with |*OB* dPSI| ≥ 0.1 and the accompanying tool-specific statistical cutoff: *Q*-value ≤ 0.05 (junctionCounts), probability_changing ≥ 0.95 (MAJIQ), *P*-value ≤ 0.05 (rMATS-turbo), adjusted *P*-value ≤ 0.05 (splAdder) or probability ≥ 0.95 (Whippet). We did not measure TPR for the dPSI evaluation because while a given event may meet the |GT dPSI| ≥ 0.1 threshold across conditions, each tool's statistical evaluation of OB dPSI measurements may justifiably call the event statistically insignificant based on numerous factors including junction read support and dispersion characteristics. To mitigate this possibility, we filtered the ground truth events for those with minimum total junction read support ≥ 15 in both conditions. For this test, TP were defined as events with either GT dPSI ≥ 0.1 and OB dPSI ≥ 0.1 or GT dPSI ≤ –0.1 and OB dPSI ≤ –0.1 (with the tool-specific statistical cutoff described earlier). FP were defined as events with |GT dPSI| < 0.02 that were called significant by a given tool. We chose this definition of FP because events with |GT dPSI| < 0.02 were a higher-confidence ‘not changing’ set of events which each tool should correctly identify as insignificant. TN were defined as events with |GT dPSI| < 0.1 that were called insignificant by a given tool. FN were defined as events with |GT dPSI| ≥ 0.1 that were called insignificant by a given tool. PPV, NPV and FDR were calculated with the described definitions of TP, FP, TN and FN. MAE was calculated as described earlier for the PSI metrics, but at cumulative |GT dPSI| thresholds.

### Analysis of interspecies and temporal alternative splicing dynamics during neuronal differentiation in primate PSCs

We analyzed RNA-seq data from human and rhesus macaque embryonic stem cells (ESCs) as well as chimpanzee and orangutan induced pluripotent stem cells (iPSCs) (39). Field *et al.* induced differentiation of the stem cells to cortical neurospheres to model prenatal brain development. Duplicate RNA-seq libraries from each time point (0, 1, 2, 3, 4 and 5 weeks of neuronal differentiation) were downloaded as compressed FASTQ files from SRA, deduplicated and mapped with STAR to the appropriate genome: GRCh38 (21), panTro4 (40), ponAbe2 (41) and rheMac8 (42) for human, chimpanzee, orangutan and rhesus macaque respectively. The GENCODE v27 basic gene annotation (21) was used as a basis for CAT (43) to generate gene annotations of similar complexity for all species. To reduce the complexity of the input transcriptomes, only basic transcripts were retained. Following this filtration, these annotations were used as input along with the mapped RNA-seq reads for StringTie v1.3.6 (16) to assemble unannotated transcripts. Using the StringTie merge command, comprehensive gene annotations were produced for each species.

To identify orthologous AS events, the whole genome sequences of human (GRCh38), chimpanzee (PanTro4), orangutan (ponAbe2), and rhesus macaque (rheMac8) were mapped to one another using minimap2 v2.11-r797 (44) with parameters *–cs* and *-asm20*. The resulting mappings were used to lift the coordinates of alternative event exons to other species with a modified version (altered such that the input BED file coordinates are semicolon-delimited rather than underscore-delimited in the name field of the output BED file) of the minimap partner utility paftools. Events were reassembled from the lifted coordinates of component exons, and checked for exon count and event type-concordance with the original event. Lifted events passing these checks were proposed as putative orthologs, and then checked against events natively identified in the target species to identify orthologous relationships. Non-one-to-one relationships were removed from consideration.

Temporal (time points 1–5 weeks of neuronal differentiation versus *t*_0_) and interspecies (paired time points compared across species) AS analyses were performed using junctionCounts. Events with |dPSI| ≥ 0.1 and *Q*-value ≤ 0.05 across conditions were considered significantly different. Events exhibiting significant splicing differences in at least one temporal comparison for each species were clustered by their temporal PSI trajectories with CLARA (cluster v2.1.6) (45) using Euclidean distance and with 500 iterations. Conservation of primate temporal splicing dynamics was assessed based on concordance with human temporal splicing dynamics with regard to PSI measurements. Genes with mean |dPSI| ≤ 0.1 for events in chimpanzee, orangutan and rhesus macaque relative to human in pairwise comparisons at each time point were categorized as genes with conserved splicing, while those with mean |dPSI| ≥ 0.3 were categorized as genes with non-conserved splicing. Gene ontology analyses for genes in the temporal event clusters and conserved and non-conserved splicing gene sets was done with Metascape (46).

Functional splicing analyses included assessment of switch events and exonic features. Switch events, which we define as events in which all transcripts consistent with one alternative form contain a particular feature while all transcripts consistent with the other form do not, were identified using cdsInsertion and findSwitchEvents (https://github.com/ajw2329/cds_insertion). In NMD switch events one form, but not the other, introduces a premature termination codon (in-frame stop codon ≥ 55 nt upstream of the final exon-exon junction when translated *in silico* from any overlapping consensus coding sequence start codon). In NSD switch events one form, but not the other, results in transcripts lacking an in-frame stop codon. In coding (to noncoding) switch events one form, but not the other, results in transcripts lacking a coding sequence. Exon ontology analysis was performed with Exon Ontology (47) using the included form coordinates of alternative exons as the test list, and the excluded form coordinates as the background.

### Data sources

RNA-seq data from mouse cerebellum and liver cells (36) and RNA-seq data from spliceostatin A and DMSO-treated HeLa cells (29) was used to generate simulated data for the benchmarking experiment. RNA-seq data and NMD target RT-PCR data from UPF1 siRNA and non-targeting siRNA-treated HEK-293 cells (30) and RNA-seq data from emetine and DMSO-treated HEK-293 T cells (31) was used to validate cdsInsertion and findSwitchEvents NMD predictions. RNA-seq data from human and rhesus macaque ESCs as well as chimpanzee and orangutan iPSCs (39) was used to demonstrate junctionCounts’ utility in a variety of applications.

## Results

### junctionCounts and its partner utilities facilitate user-friendly isoform-level analysis

junctionCounts can characterize alternative events in any user-provided transcriptome annotation. It defines the minimal set of binary splicing events that distinguish alternative isoforms within gene models and classifies events into event types that range from simple, canonical events to complex events that capture coordinated splicing of multiple event features comprising multiple event types. To be clear, in the context of this work, we define complex events as those involving multiple alternative feature types (i.e. a CF event representing coordinated AF-SE-A3 splicing), which excludes MS events, for example. Other AS analysis tools, including MAJIQ (32) and Whippet (20), can also characterize complex events. However, junctionCounts uniquely summarizes complex event junction read support within an easily interpretable binary context; assigning a single PSI value to the included and excluded form of events rather than to individual splice sites or features. Beyond alternative event definition and classification, junctionCounts’ partner utilities enable valuable prediction of functional outcomes, including NMD, by connecting individual splicing events to their effects on the CDS at the isoform level. cdsInsertion derives CDS information from a transcriptome annotation and findSwitchEvents uses junction coordinate keys to associate the included and excluded form of events to their respective alternative isoforms. Altogether, junctionCounts presents an easy to install, easy to use set of tools with relatively few dependencies and the novel capability of event-to-isoform CDS characterization within the AS analysis milieu.

### junctionCounts accurately quantifies alternative splicing events

We evaluated junctionCounts’ performance on simulated data, with known ground truth PSI values for junctionCounts-defined events, modeled on real RNA-seq data with that of four established AS analysis tools: MAJIQ (32), rMATS-turbo (33), splAdder (34) and Whippet (20). We generated four simulated datasets in total: three datasets at 25, 50 and 75 million reads per library were modeled on mouse cerebellum and liver RNA-seq data (32) with triplicates per cell type to evaluate performance at different library sizes. The fourth dataset was modeled on human RNA-seq data at 50 million reads per library with triplicates for two conditions: spliceostatin A (SSA) and DMSO treatment. This dataset provided a larger pool of events to test relative to the murine datasets, including over 2000 MR events of which there were <100 in the murine datasets. Each tool was run on a laptop, as described in the Materials and Methods, and the time from start to finish of all analysis steps on all 6 samples of each dataset and the peak memory cost at any point during that time were recorded (Figure 2A, B). junctionCounts exhibited the median time and memory cost among the other tools, which scaled with library size and transcriptome complexity for all tools.

Each publicly available AS analysis tool identifies and quantifies AS events within its own event type repertoires and definitions, thus complicating their comparison. rMATS-turbo and splAdder are limited to non-terminal event types, while junctionCounts, MAJIQ and Whippet are each capable of characterizing terminal events and additionally non-canonical, complex event types involving coordinated splicing of multiple alternative feature types. Each tool's set of events identified from the simulated data were matched to junctionCounts-defined events to compare ground truth to observed PSI values. In order for an event, identified by a given tool, to match with a junctionCounts event, the coordinates of its participating features each had to overlap by ≥ 95%, which provides latitude for minute variation of event exon/intron nodes across tools. Despite this flexibility, no complex events across junctionCounts, MAJIQ or Whippet met the requirements for comparison, which reflects each tool's unique approach to event definition, even with canonical event types. The mean overlap of junctionCounts events with other tools across the four simulated datasets was: 78% for MAJIQ, 75% for rMATS-turbo, 51% for splAdder and 13% for Whippet – amounting to thousands of events per tool. Each tool was tested individually on the subset of junctionCounts-defined events they approximately reproduced (Figure 2C, Supplemental Figure S1A–E).

We first measured performance at the PSI level (in-depth descriptions of testing procedures in Materials and Methods). At the PSI level, we evaluated each tool's event detection and quantification capabilities. Here, we defined sensitivity (true positive rate; TPR) as the proportion of events detected that had both a measured and ground truth PSI > 0. In this context, the sensitivity test measured a tool's ability to correctly assign read support to an event rather than its ability to reproduce ground truth events. Next, we tested false discovery rate (FDR) as the proportion of events that had a measured PSI ≥ 0.05 and a ground truth PSI < 0.05 relative to all events with a measured PSI > 0. This threshold was applied because each tool commonly misattributed miniscule PSI values to events with ground truth PSI 0, which in most normal use cases isn’t a problem as final results are typically filtered for events meeting a minimal PSI value among conditions or replicates. Instead, this FDR test quantified the rate at which tools misattributed read support to a substantial degree (≥0.05 PSI) that would be problematic in typical AS analysis settings. Finally, we tested the accuracy of true positive event (measured and ground truth PSI > 0) quantification in terms of mean absolute error (MAE). junctionCounts generally represented the median of the five tools across these metrics (Figure 2D–G), with the caveat that it was tested on the largest number of events and event types (Supplemental Figure S1A-E). When stratified by event type, we found that junctionCounts consistently maintains a FDR < 3% and the largest area under curve of cumulative mean absolute error distribution relative to the other tools for AF, AL, MX and MS event types among those directly compared (Supplemental Figure S1F–I).

Next, we assessed performance at the event change (dPSI) level, focusing on each tool's accuracy in calling and quantifying significant and insignificant event changes at cumulative dPSI thresholds. We measured positive predictive value (PPV), which is the proportion of correct significant event calls made by a given tool that have |ground truth dPSI| ≥ 0.1 relative to all significant event calls and negative predictive value (NPV), or the proportion of correct insignificant event calls with |ground truth dPSI| < 0.1 relative to all insignificant event calls. We also measured FDR, which we defined as the proportion of incorrect significant event calls with |ground truth dPSI| < 0.02 among all significant calls, and finally the MAE of measured vs. ground truth dPSI values. junctionCounts had the highest dPSI Pearson correlation coefficient, just under 0.8, among the other tools on the murine datasets, and notably never surpassed 0.1 dPSI MAE at any dPSI threshold, while Whippet, rMATS-turbo and splAdder all surpassed 0.3 dPSI MAE on the SSA dataset. Whippet and junctionCounts maintained the smallest distance between cumulative PPV/NPV and FDR curves across the 75M and SSA datasets, while junctionCounts achieved the minimal distance of cumulative MAE curves among the other tools across datasets (Figure 2H–K). Taken together, junctionCounts had the most consistent performance across dPSI metrics between murine and human datasets compared to the other tools.

### Characterizing temporal and species-specific alternative splicing dynamics during primate neuronal differentiation

**Figure 3.**
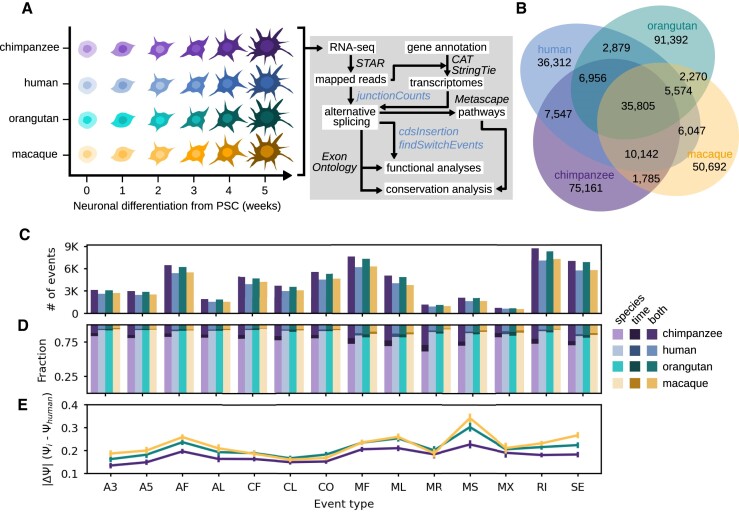
Application of junctionCounts to a primate neuronal differentiation time course experiment. (**A**) Schematic of five-week chimpanzee, human, orangutan and rhesus macaque neuronal differentiation from pluripotent stem cells and subsequent RNA-seq analysis workflow. (**B**) Venn diagram of the events identified by junctionCounts in each primate transcriptome. (**C**) The total number of significant events (|dPSI| ≥ 0.1 and *Q*-value ≤ 0.05 in at least one temporal or interspecies comparison) by event type for each species. (**D**) The fraction of events that were significantly different across species, time or both factors. (**E**) Evaluation of conserved splicing by event type, measured by |dPSI| against human PSI values.

After establishing that junctionCounts competently characterizes AS in simulated data, we next wanted to examine its utility on real data. To that end, we analyzed a primate neuronal differentiation RNA-seq dataset comprising human and rhesus macaque ESCs as well as chimpanzee and orangutan iPSCs (39) (Figure 3A). We hypothesized that because the four primates share 90–99% genome sequence conservation (48), junctionCounts should identify a substantial number of orthologous AS events across the four primates (49). We further expected to observe substantial species-specific splicing dynamics during neuronal differentiation as previous interprimate studies have reported (49).

We used CAT (43) on the GENCODE v27 (21) basic gene annotation to generate gene annotations of similar complexity for all species. We then used StringTie v1.3.6 (16) on the resultant gene annotations along with the mapped RNA-seq reads to assemble unannotated transcripts. Thus we produced comprehensive gene annotations for each species. Using junctionCounts, we identified approximately 143K, 111K, 151K and 113K possible events in the chimpanzee, human, orangutan and rhesus macaque gene annotations respectively. And to identify orthologous AS events, we performed pairwise mapping of the whole genome sequences of human (GRCh38), chimpanzee (PanTro4), orangutan (ponAbe2), and rhesus macaque (rheMac8) using minimap2 (44). Using the mappings, we lifted the coordinates of alternative event exons to other species using paftools. We then reassembled events from the lifted coordinates of component exons, assessed exon count and event type-concordance with the original events and checked these against events identified in the target species to establish orthologous relationships for which only one-to-one relationships were considered.

In all pairwise interspecies event set comparisons, at least 40% of events were not species-specific, with over 35K orthologous events common to all four primates (Figure 3B). We next quantified these events with junctionCounts which uses junction reads from the mapped RNA-seq data, after which we performed event-level – statistically tested with DEXSeq v3.19 (27) – pairwise temporal comparisons (week_*i*_ versus week_0_ of neuronal differentiation) and interspecies comparisons (between corresponding time points; week_*i*_ versus week_*i*_) with duplicates per condition. We identified 61K, 50K, 59K and 51K events that were significantly differentially spliced (|dPSI| ≥ 0.1 and *Q*-value ≤ 0.05) in at least one temporal or interspecies comparison in chimpanzee, human, orangutan and rhesus macaque respectively. We observed that the majority of splicing changes were in interspecies comparisons (Figure 3D), with RI, MF, SE and AF constituting the most commonly differentially spliced event types (Figure 3C). Intriguingly, when we compared orthologous event PSI values by event type between each primate and human across corresponding time points—as a proxy for conservation of splicing dynamics – we found that complex event types (CF, CL and CO) displayed the closest central tendency of PSI values to those of human cells (Figure 3E). This finding may lend credence to the value of characterizing complex event types and their involvement in primate neuronal differentiation.

### junctionCounts uncovers novel splicing dynamics in genes relevant to neuronal differentiation and function

**Figure 4.**
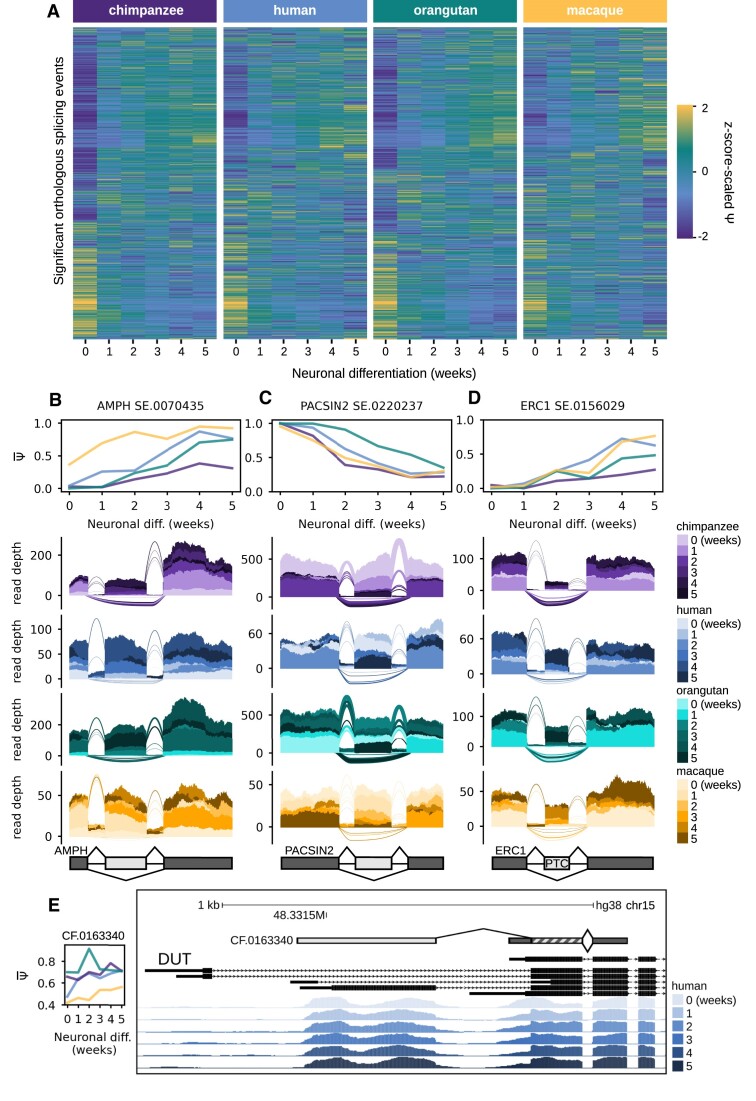
junctionCounts uncovers conserved and species-specific temporal splicing patterns among orthologous splicing events across the four primates. (**A**) Heatmap of *Z*-score-scaled PSI values for significant orthologous splicing events (|dPSI| ≥ 0.1 and *Q*-value ≤ 0.05 in at least 1 temporal or interspecies comparison) with each row corresponding to the same event across all four primates. (**B**) Mean PSI trajectories and RNA-seq coverage at a skipped exon event in AMPH with species-specific temporal splicing patterns across chimpanzee, human and rhesus macaque. (**C**) Mean PSI trajectories and RNA-seq coverage at a skipped exon event in PACSIN2 with a conserved temporal splicing pattern. (**D**) Mean PSI trajectories and RNA-seq coverage at an PTC-containing skipped exon event in ERC1 with a conserved temporal splicing pattern. (**E**) UCSC Genome Browser snapshot of human read support at a complex first exon event in DUT, which measures the inclusion of one of several distal alternative first exons and its subsequent second exon versus the proximal first exon which overlaps with the alternative second exon. The subpanel to the left shows the mean PSI of the included form at each time point of neuronal differentiation in each primate. In panels (B)–(D), the included form of the alternative event contains both the dark and light gray components, while the excluded form only contains the dark gray components. In panel (**E**), the same is true except the included form does not contain the dark gray fragment at the 5′ end of the central exon. In panels (B)–(E), the upright and inverted arches represent junction read coverage for the included and excluded form respectively.

Among the 17K significant events (|dPSI| ≥ 0.1 and *Q*-value ≤ 0.05 in at least one comparison) that were orthologous in all four primates (Figure 4A), we hypothesized that junctionCounts would both recapitulate previously reported splicing phenomena and identify novel events in genes involved in neuronal differentiation and function. Here, we highlight several such findings. Amphiphysin 1 (AMPH) and Amphiphysin 2 (BIN1) are both enriched in the mammalian brain and participate in synaptic vesicle endocytosis (50,51). Splice variants of BIN1 have been reported in the brain as well as other tissue types (43), but the implications of AS in AMPH1 remain unexplored. We report a SE event involving exon 17 of AMPH1 (Figure 4B), which is the only scenario of AS that affects the CDS among AMPH1 isoforms annotated in GENCODE V44. According to Exon Ontology (47), AMPH1 exon 17 encodes an intrinsically unstructured polypeptide region which contains an *O*-phospho-l-serine modification site. This exon is increasingly spliced in over the time course with species-specific trajectories and magnitudes, possibly indicating a functional role for AMPH1 exon 17 inclusion in neurons.

Protein kinase C and casein kinase II substrate in neurons 2 (PACSIN2) is the only known member of the PACSINs whose expression isn’t cell type-specific, in humans. All three PACSINs have been reported to play a role in trafficking AMPA receptors in and out of synapses, which is a crucial factor in important neuronal processes including synaptic transmission and plasticity (52,53). We identified a SE event involving exon 9 of PACSIN2 for which the included form uniformly decreases from the dominant to the minor form over the course of neuronal differentiation (Figure 4C). Similarly to the aforementioned SE event in AMPH1, this SE event is the only CDS-altering event among PACSIN2 isoforms annotated in GENCODE V44. According to the GTEx V8 RNA-Seq Read Coverage by Tissue track on the UCSC Genome Browser (54), PACSIN2 exon 9 inclusion is dominant in all non-neuronal tissue types, while the excluded form is dominant in 9 out of 14 neuronal tissue types. These observations make a compelling case for neuron-specific AS of PACSIN2, resulting in the preferential exclusion of exon 9 in several neuronal cell types.

We discovered a conserved ERC1 PTC-inducing SE event involving exon 18 in isoform ENST00000355446.9 (in GENCODE V44), that to our knowledge has not been previously reported by other groups (Figure 4D). Inclusion of this exon may produce an NMD substrate, but could potentially yield a functional protein isoform at least 30 residues shorter at the C-terminus relative to isoforms consistent with the excluded form. ERC1 has been described to undergo neuron-specific AS and is implicated in important functions including neurotransmitter release and neuronal differentiation (55,56). Over the five week course of neuronal differentiation, the PTC-inducing SE event follows a consistent pattern of becoming increasingly spliced in across the four primates. Interestingly, AS at the C-terminus of Erc1 in rats was shown to generate two isoforms: Erc1a and Erc1b. The latter of which is the brain-specific, shorter isoform that alone can bind to presynaptic active zone proteins, called RIMs, that regulate neurotransmitter release (57). Taken together, these observations suggest a potential functional role for the inclusion of the ERC1 poison exon in differentiating neurons.

Deoxyuridine 5′-triphosphate nucleotidohydrolase (DUT) is an important enzyme involved in genome integrity maintenance that prevents uracil misincorporation into DNA. DUT expression has been shown, through knockout studies, to be essential to embryonic development and especially to later stages of differentiation in mice (58). We identified a CF event in DUT (Figure 4E), in which the included form corresponds to the DUT-M isoform and the excluded form corresponds to the DUT-N isoform (59). The DUT-M isoform localizes to mitochondria via a mitochondrial targeting presequence located in the first exon consistent with the included form of the CF event and is expressed constitutively. The DUT-N isoform localizes to the nucleus and its expression is induced during the G_0_ to S phase transition. Exit from the cell cycle into G_0_ phase triggers DUT-N protein degradation. Thus, DUT-N isoform expression is tightly linked to nuclear DNA replication (59). We observed that the CF event is increasingly spliced in – meaning DUT-M isoform expression gradually eclipses DUT-N isoform expression over the time course – which is to be expected as the primate pluripotent stem cells (PSCs) progressively commit to neuronal cell fates with decreasing cell cycle activity (60). These findings demonstrate that junctionCounts can handily uncover novel splicing phenomena.

### Temporal regulation of alternative splicing directs the transition from pluripotent to neuronal cell fate

**Figure 5.**
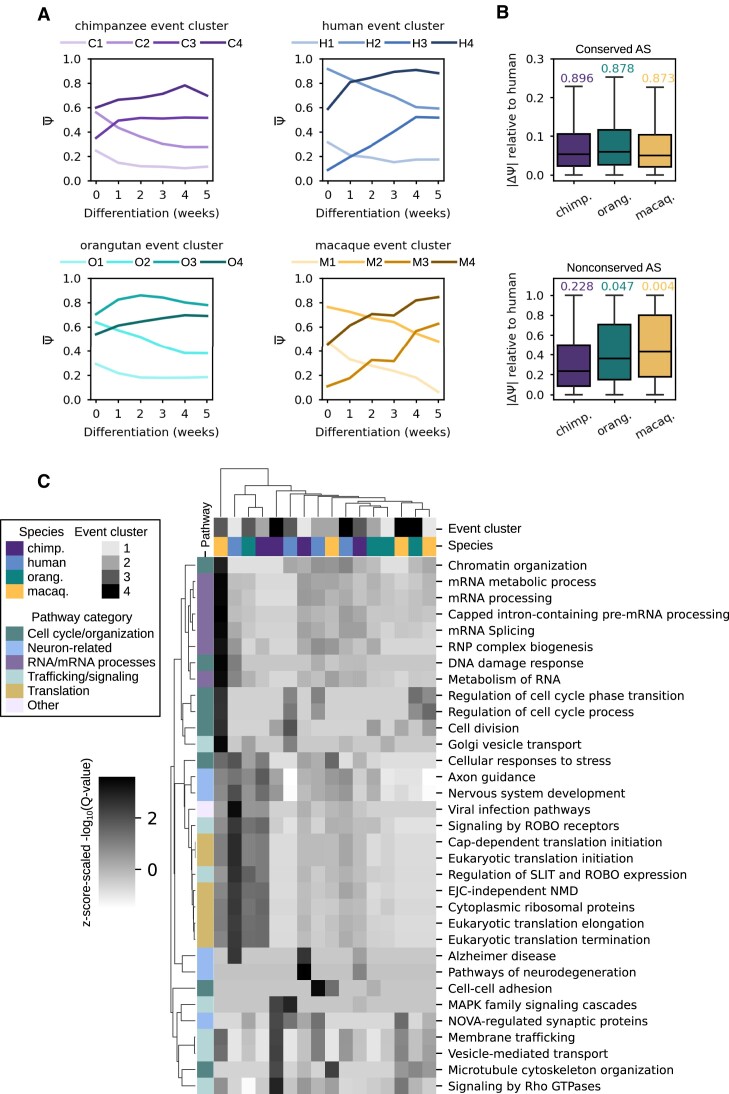
Gene ontology analyses for grouped event sets by temporal PSI trajectories. (**A**) Four gene-level clusters derived with CLARA from temporal expression trajectories for each species. (**B**) Genes with mean |dPSI| ≤ 0.1 for events in chimpanzee, orangutan and rhesus macaque relative to human were categorized as genes with conserved splicing. Boxplots showing the distribution of each primate's per-gene mean |dPSI| relative to human (upper subpanel) with Pearson correlation coefficient of species-specific PSI values against human PSI values above them. The same for genes with nonconserved splicing based on mean |dPSI| ≥ 0.3 (lower subpanel). (**C**) Heatmap of *Z*-score-scaled-log_10_(*Q*-value) of Metascape (38) pathway enrichment in temporal event clusters.

High levels of AS and cell type-specific isoform expression are observed in neurons and during neuronal differentiation (7,61). We postulated that genes exhibiting dynamic temporal splicing would be enriched for neuronal biological pathways. Taking the subset of significant AS events (|dPSI| ≥ 0.1 and *Q*-value ≤ 0.05 in at least one temporal comparison), we generated the four most distinct clusters of events based on the Euclidean distance of their temporal PSI trajectories using CLARA (45) for each species (Figure 5A). Additionally, we identified subsets of genes with conserved (mean |dPSI| ≤ 0.1 for all events per gene) and nonconserved (mean |dPSI| ≥ 0.3 for all events per gene) splicing patterns in chimpanzee, orangutan and rhesus macaque relative to human in pairwise comparisons at each time point (Figure 5B). We then used Metascape (46) to identify enriched biological pathways in the sets of genes from each cluster of temporally regulated events (Figure 5C) and for the conserved and nonconserved splicing gene sets (Supplementary Figure S2).

Four similar but distinct event clusters were identified in each primate. Across the primates, cluster 1 (chimpanzee, human, orangutan and rhesus macaque corresponding to C1, H1, O1 and M1 respectively) generally represents alternative features (exons, introns, splice sites, etc.) whose inclusion is marginal in PSCs and declines over the course of differentiation. Cluster 2 (C2, H2, O2 and M2) represents alternative features whose inclusion is dominant in PSCs and declines during differentiation. Pathways similarly enriched in clusters H1, M1 and C2 indicate the preferred exclusion of particular alternative features in the mature splicing program of genes related to: translation, NMD, axon guidance and nervous system development. Cluster 3 (C3, H3, O3 and M3) generally contains alternative features whose inclusion is marginal in PSCs and increases during differentiation, while cluster 4 (C4, H4, O4 and M4) contains dominantly included alternative features that further increase until peaking at week 4 during differentiation. Clusters H3 and O3 indicate increasing inclusion of alternative features in the mature splicing program of genes related to: NOVA-regulated synaptic proteins, axon guidance and nervous system development. Cluster C3 indicates a slight increase in alternative feature inclusion in genes related to mRNA processing and translation. The conserved splicing event set was enriched for genes in critical pathways including cell cycle processes, signaling, AS, and interestingly, in neurodegeneration pathways. The subset of complex conserved splicing events (CF, CL and CO) was enriched for nearly all the same pathways, revealing the prevalence of complex events in important pathways (Supplementary Figure S2). For example, we identified a conserved CO event in NCKAP1, which is involved in Rho GTPase signaling, and a conserved CL event in QKI, which is involved in pre-mRNA processing and AS (Supplementary Figure S3). Taken together, these results highlight the intricate temporal regulation of splicing as PSCs develop into neuronal cells and shed light on the biological relevance of species-specific and conserved splicing dynamics.

### Emergent alternative features underlie many instances of species-specific alternative splicing

**Figure 6.**
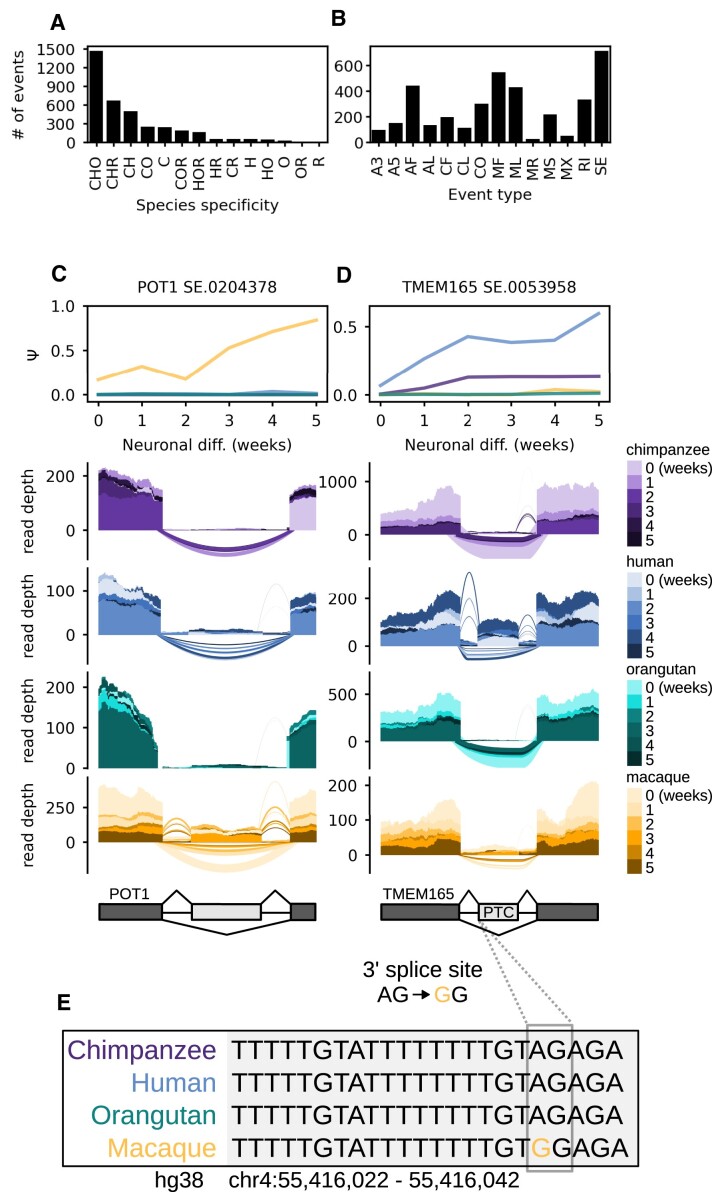
Emergent/species-specific alternative feature usage. (**A**) Barplot showing the number of species-specific events (min(PSI) ≥ 0.05 in a given species) exhibiting significant temporal regulation (|dPSI| ≥ 0.1 and *Q*-value ≤ 0.05 in at least one temporal comparison). ‘C’, ‘H’, ‘O’ and ‘R’ are abbreviations for chimpanzee, human, orangutan and rhesus macaque respectively. Combinations of these abbreviations represent instances of events that meet the min(PSI) threshold in a set of species and are significantly temporally regulated in at least 1 species in the subset. (**B**) Barplot displaying the same set of species-specific events as in (A) but stratified by event type instead of species. (**C**) Mean PSI trajectories and RNA-seq coverage at a rhesus macaque-specific skipped exon event in POT1. (**D**) Mean PSI trajectories and RNA-seq coverage at a human-specific skipped exon event in TMEM165. (**E**) Macaque-specific point mutation just upstream of the 3′SS of the TMEM165 skipped exon event shown in (**D**).

Because we observed that some events had miniscule or zero PSI values in particular species, we hypothesized that a subset of the aforementioned nonconserved event set represents events that sufficiently map (sequence divergence ≤ 20%) pairwise between all four primate genomes but contain alternative features that are only used (included) by specific primates despite the apparent presence of splice site and branch site sequences. We call features used by specific species emergent alternative features, potentially indicating exonization events. Indeed, we identified 3753 events, in 1922 genes, exhibiting significant temporal regulation (|dPSI| ≥ 0.1 and Q-value ≤ 0.05 in at least one temporal comparison) while having a min(PSI) ≥ 0.05 in only a subset of the four primates (Figure 6A). Of these events, the most prominent event types were SE, MF, AF and ML (Figure 6B). Protection of Telomeres 1 (POT1) is an example of a rhesus macaque-specific SE event in the 5′UTR (Figure 6C). This SE event is likely an instance of species-specific differences in exon induction related to neuronal differentiation, as the alternative exon is annotated in GENCODE V44 and exhibits cell type-specific expression in a number of human neuronal tissues according to GTEx V8 RNA-Seq Read Coverage by Tissue despite its lack of inclusion in our human samples. Transmembrane Protein 165 (TMEM165) is an example of a human-specific PTC-containing SE event that is potentially the product of Alu exonization (62), as it overlaps an antisense AluJb element (Figure 6D). Furthermore, one piece of evidence that suggests that it may be a bona fide emergent alternative cassette exon is that the rhesus macaque genome sequence has an A-to-G mutation 3 nt upstream of the 3′SS while the other three primates have a canonical 3′SS dinucleotide (Figure 6E). In short, identification of AS events in orthologous sequences between species may be an effective approach to uncover potential emergent alternative features.

### cdsInsertion and findSwitchEvents connect alternative splicing events to potential functional impacts

An enduring problem in the study of AS is the challenging nature of connecting events to functional impacts, whether at the mRNA or protein level. We used cdsInsertion to annotate transcripts with information regarding the lengths of the UTRs and CDS, the presence of potential PTCs and other details gleaned from overlapping annotated start codons. Next, we employed findSwitchEvents to couple isoform-level CDS information to junctionCounts-defined events to identify ‘switch events’, which are instances in which a particular property is exclusive to transcripts consistent with the included or excluded form. We propose that this approach enables users to connect AS events to functional outcomes, comprehensively profile switch event regulation and to discover novel instances of NMD/NSD.

**Figure 7.**
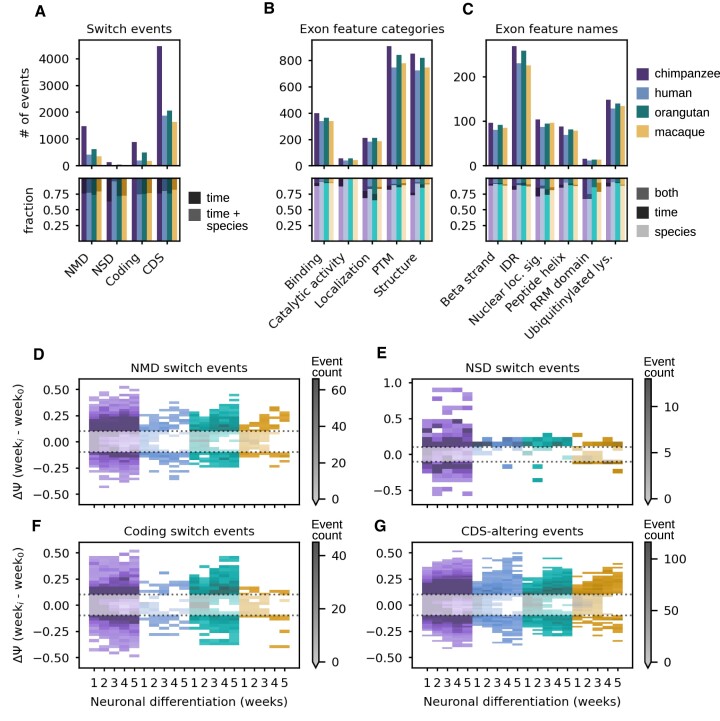
Analysis of splicing events with potential functional impacts to mRNA stability, coding capacity or protein function. (**A**) Total number of significant splicing events (|dPSI| ≥ 0.1 and *Q*-value ≤ 0.05 in at least one temporal or interspecies comparison) that are predicted to: induce nonsense-mediated decay (NMD), induce non-stop decay (NSD), break the open reading frame (coding switch) or alter the coding sequence (CDS). (**B**) Exon feature category support for significant event coordinates overlapping exon features in the Exon Ontology database. (**C**) Specific exon features affected by significant events, indicating potential impacts to structural or functional protein elements. The fraction of events in (A)–(C) that were significantly different across species, time or both factors (lower subpanels). (**D**) Bivariate histogram showing the distribution of NMD switch event dPSIs over the time course of neuronal differentiation relative to pluripotent stem cells (week 0) in each primate. (**E**)–(**G**) show the same for NSD switch, coding switch and CDS-altering events, respectively.

Among significant events (|dPSI| ≥ 0.1 and *Q*-value ≤0.05 in at least one comparison), we identified hundreds of events predicted to confer NMD, NSD and coding-to-noncoding switches as well as >1600 CDS-altering events in each species (Figure 7A). To investigate the potential structural and functional impacts of CDS-altering events, we mapped event coordinates to protein features with Exon Ontology (47). The five protein feature categories most frequently overlapping alternative exons were: post-translational modification (PTM), structure, binding, localization and catalytic activity (Figure 7B). Interestingly, intrinsically disordered regions (IDRs) were the most highly represented feature (Figure 7C). In agreement with these findings, IDRs have been described as preferred loci for both AS and PTMs (63,64).

At the mRNA-level, NSD and NMD substrates are expected to be degraded via translation-dependent pathways to prevent the production of potentially harmful truncated proteins (65). However, in certain contexts expression and translational activation of NMD substrates can play important roles in biological functions, including in neuronal differentiation (66). Another possible outcome of AS is the generation of noncoding transcripts from protein coding genes (67). We characterized the regulation of these three phenomena during neuronal differentiation and found that NMD and coding-to-noncoding switch events became progressively more differentially spliced over the course of neuronal differentiation relative to PSCs (Figure 7D–F). NSD switch events were relatively rare, but they were surprisingly overrepresented in chimpanzee relative to the other primates (Figure 7E). Overall, we did not observe a monotonic increase in NMD substrate abundance during neuronal differentiation and we found that NMD/NSD/coding-to-noncoding switch events generally followed species-specific temporal trajectories (Supplementary Figure S4). CDS-altering events were also increasingly differentially spliced over the course of differentiation (Figure 7G), likely owing to the gradual definition of a mature splicing program that requires cell-type specific expression of isoforms with distinct functions (68).

## Discussion

This paper describes our efforts to develop an accurate, rigorous, easy to use and interpret alternative splicing analysis tool capable of identifying and quantifying a comprehensive repertoire of splicing event types, with the addition of novel capabilities. junctionCounts accurately recapitulates ground truth PSI and dPSI values from simulated data and performed well in our benchmarking experiment against MAJIQ (36), rMATS-turbo (33), splAdder (34) and Whippet (20) (Figure 2, Supplemental Figure S1). It identifies and accurately quantifies a wide array of event types including complex event types that represent coordinated splicing of multiple alternative features. In contrast to MAJIQ, rMATS-turbo, splAdder and Whippet, junctionCounts identifies events from a gene annotation alone, eliminating the need to generate new splice graphs/event dictionaries for each individual dataset. Contrarily, the other tools use information from mapped RNA-seq data during splice graph generation, so event identification scales directly with library read depth and can include novel splice junctions. Novel splice junction detection can be achieved with junctionCounts by providing a transcriptome assembled from RNA-seq reads using a tool like StringTie (16). In terms of its novel capabilities, tools like vast-db (69) and IsoformSwitchAnalyzeR (70) provide valuable insights into the functional consequences of AS events, while junctionCounts (in concert with its partner utilities: cdsInsertion and findSwitchEvents) is unique in its ability to predict event-level functional consequences including NMD, NSD and coding-to-noncoding switches in an annotation-agnostic manner that is applicable to any given dataset. We show through the analysis of published UPF1-knockdown (29,30) and emetine (31) human RNA-seq data that junctionCounts accurately predicts NMD switch events (Figure 2L–N).

After rigorously testing junctionCounts and its partner utilities, we applied them to a primate neuronal differentiation RNA-seq dataset comprising human and rhesus macaque ESCs as well as chimpanzee and orangutan iPSCs (39). We identified 50–61K significant splicing events (|dPSI| ≥ 0.1 and *Q*-value ≤ 0.05 in at least one temporal or interspecies comparison) in each species, with 17K significant orthologous events across all four primates (Figure 4A). Within these orthologous events, junctionCounts recapitulated previously reported splicing phenomena (59) and identified previously unreported events in several genes relevant to neuronal differentiation (Figure 4B–E). RT-PCR experiments were used to verify some of these events, including SE events in GABBR1 and MYCBP2 in human and macaque cells (Supplementary Figure S5). We additionally clustered events by their temporal splicing dynamics, uncovering distinct event trajectories that capture the tight regulation of splicing during development (Figure 5A). Highly relevant biological pathways were represented in these event clusters, including axon guidance, nervous system development and SLIT/ROBO signaling. Pathways in chromatin organization, mRNA processing and cell cycle processes were also shown to undergo and potentially underlie splicing regulation (Figure 5C). Within the set of events with nonconserved splicing patterns, we uncovered thousands of events containing alternative features that were used in some primates but not in others, suggesting potential emergent alternative features (Figure 6). Lastly, we used cdsInsertion and findSwitchEvents to connect events to predicted NMD/NSD/coding-to-noncoding switches based on isoform-level CDS properties exclusive to their included or excluded forms. This allowed us to profile temporal NMD/NSD regulation (Figure 7D-E) and to identify potential NMD substrates (Figures 4D and 6D). Altogether, we exhibited the functionality of junctionCounts in a variety of analysis contexts and presented its application to the characterization of splicing in evolution, neuronal differentiation and NMD.

Besides this work, we further note that several of our colleagues have already implemented and published results using a beta version of junctionCounts. These studies include a variety of model systems such as human and non-human primate cell lines, *C. elegans*, and yeast. Suzuki *et al.* looked at the effects of KIN17 and PRCC mutations on 5′ and 3′SS usage during development in *C. elegans*. They found both direct and potentially indirect changes in alternative 5′ and 3′SS usage, some of which were related to developmental and population dynamics. They additionally RT-PCR-verified a number of these events to differentiate between embryonic-type splicing and somatic-type splicing (71). Cartwright-Acar et al. (2022) characterized splicing changes in the presence of class II suppressors of uncoordination in an *unc-73(e936)* mutant forward genetic screen in *C. elegans*. They found that the majority of alternative 5′SS usage changes were in introns containing true alternative 5′SS and that suppressors rarely activated novel cryptic alternative 5′SS. They further RT-PCR verified several of the alternative 5′SS and 3′SS events, and finally asserted that the class II suppressors they studied may work at mutually exclusive stages of spliceosome assembly or use different mechanisms to maintain 5′SS identity based on their ability to differentiate between alternative 5′ splicing events that are unique to particular suppressors (72). Draper *et al.* quantified events across polyribosome fractions and between primates to assess the conservation of alternative splicing coupled to translational control (ASTC). They identified subsets of alternative events with either conserved or species-specific sedimentation profiles and discovered that alternative exons with conserved sedimentation had higher sequence conservation relative to those with species-specific sedimentation. They additionally tested three ASTC SE events using translational luciferase reporters (73). Hunter *et al.* examined the effect of splicing inhibitors on intron splicing efficiency in *S. cerevisiae*. They found that individual introns had distinct sensitivities, including during co-transcriptional splicing, to different splicing inhibitors. Interestingly, they found that yeast sequences including the branch point consensus motif contribute to the differences in sensitivity (74). Osterhoudt *et al.* explored changes in 3′SS usage upon SACY-1 perturbation in introns with pairs of 3′ splice sites ≤ 18 nucleotides away from each other. They found that both SACY-1 depletion and a SACY-1 mutation lead to a clear unidirectional increase in proximal alternative 3′SS usage, which they RT-PCR-verified for several events (75). Collectively, our collaborators found junctionCounts easy to implement and show, through these works, its capacity to generate high quality results.

Beyond its flexibility and user-friendliness, junctionCounts stands out as a useful approach because it identifies both canonical and non-canonical alternative events. Many tools are limited to non-terminal and/or relatively rudimentary event types. The few that characterize complex or non-canonical event types are difficult to interpret. junctionCounts utilizes the concept of binary alternative events; identifying clear instances of the inclusion and exclusion of alternative features. This concept is well-established and pervades the splicing literature. It remains popular because binary alternative events can be accurately quantified relative to full-length transcripts, they likely accurately represent (a subset of) transcript structure as compared with full-length transcripts, and they exclude gene segments not relevant to the regulation of the event (i.e. introns and exons outside and distal to the event). The first two reasons will likely decrease in validity as improving long-read sequencing approaches provide more accurate representations of the ground-truth expressed transcriptome. The biggest problem may lie with the third reason, considering that the contribution of factors not necessarily local to an event itself can be important to its regulation (76,77).

At present, however, focusing on the local site of alternative events affords the opportunity to consider the behavior of hundreds or thousands of similar events and to look for trends in features that may explain their behavior. However, a key weakness of the traditional binary event is the existence of loci in which more than two alternative sub-transcripts overlap and are subject to simultaneous changes in relative abundance. While such non-binary events could be represented as the collection of binary events involving all possible pairs of sub-transcripts, this representation loses information as the regulatory decision is likely to be made in the context of all possibilities. A number of efforts such as MAJIQ (32) and Whippet (20) have attempted to address this issue with several approaches. Nonetheless, junctionCounts presents a step in the right direction by characterizing events that don’t fit into canonical binary event definitions.

Lastly, junctionCounts’ main innovation lies in its ability to bridge the gap between event-level and isoform-level analysis with regard to the implications of AS events on transcript coding and translational capacity, via cdsInsertion. In an ideal case, studies that intend to consider translation and its implications on a transcriptome-wide scale would include an experimental technique to empirically define CDS regions or start codons. For example, ribosome profiling and approaches like TI-seq (78) can serve as a basis for empirically defining whole CDS or translation start sites respectively. However, as such data are typically unavailable due to the additional cost and complexity of these approaches, tools like cdsInsertion are useful. cdsInsertion fleshes out the putative characteristics of unannotated transcripts by performing *in silico* translation from known overlapping start codons, and thus permits the development of hypotheses to explain properties imparted by AS. Its partner tool, findSwitchEvents, infers alternative event characteristics from those of its constituent isoforms. Altogether, junctionCounts, cdsInsertion and findSwitchEvents comprise a method for the accurate characterization of AS and the novel capacity to couple events to potential functional outcomes.

## Supplementary Material

lqae093_Supplemental_File

## Data Availability

RNA-seq data from mouse cerebellum and liver cells (36) used to generate simulated data for the benchmarking experiment: publicly available at the NCBI GEO (Accession no.: GSE54652). RNA-seq data from spliceostatin A and DMSO-treated HeLa cells (29) used to generate simulated data for the benchmarking experiment: publicly available in the ArrayExpress database (Accession no.: E-MTAB-6060). RNA-seq data and NMD target RT-PCR data from UPF1 siRNA and non-targeting siRNA-treated HEK-293 cells (30) used to validate cdsInsertion and findSwitchEvents NMD predictions: publicly available at the NCBI GEO (Accession no.: GSE176197). RNA-seq data from emetine and DMSO-treated HEK-293 T cells (31) used to validate cdsInsertion and findSwitchEvents NMD predictions: publicly available at the NCBI GEO (Accession no.: GSE89774). RNA-seq data from human and rhesus macaque ESCs as well as chimpanzee and orangutan iPSCs (39): publicly available at the NCBI GEO (Accession no.: GSE106245). The version of junctionCounts and partner utilities used in this study are published on Zenodo (https://doi.org/10.5281/zenodo.11186192). junctionCounts and its partner utilities are also available on GitHub: https://github.com/ajw2329/junctionCounts and https://github.com/ajw2329/cds_insertion.

## Notes

Present address: Jeremy Sanford, Department of Molecular, Cell and Developmental Biology, University of California Santa Cruz, Santa Cruz, CA 95064, USA.
