# Supplementary material for: junctionCounts: comprehensive alternative splicing analysis and prediction of isoform-level impacts to the coding sequence
Source: NAR Genom Bioinform. 2024 Aug 9;6(3):lqae093. doi: 10.1093/nargab/lqae093 (PMC11310779; doi:10.1093/nargab/lqae093)
Supplement: lqae093_Supplemental_File [file lqae093_supplemental_file.pdf]

Fig S1

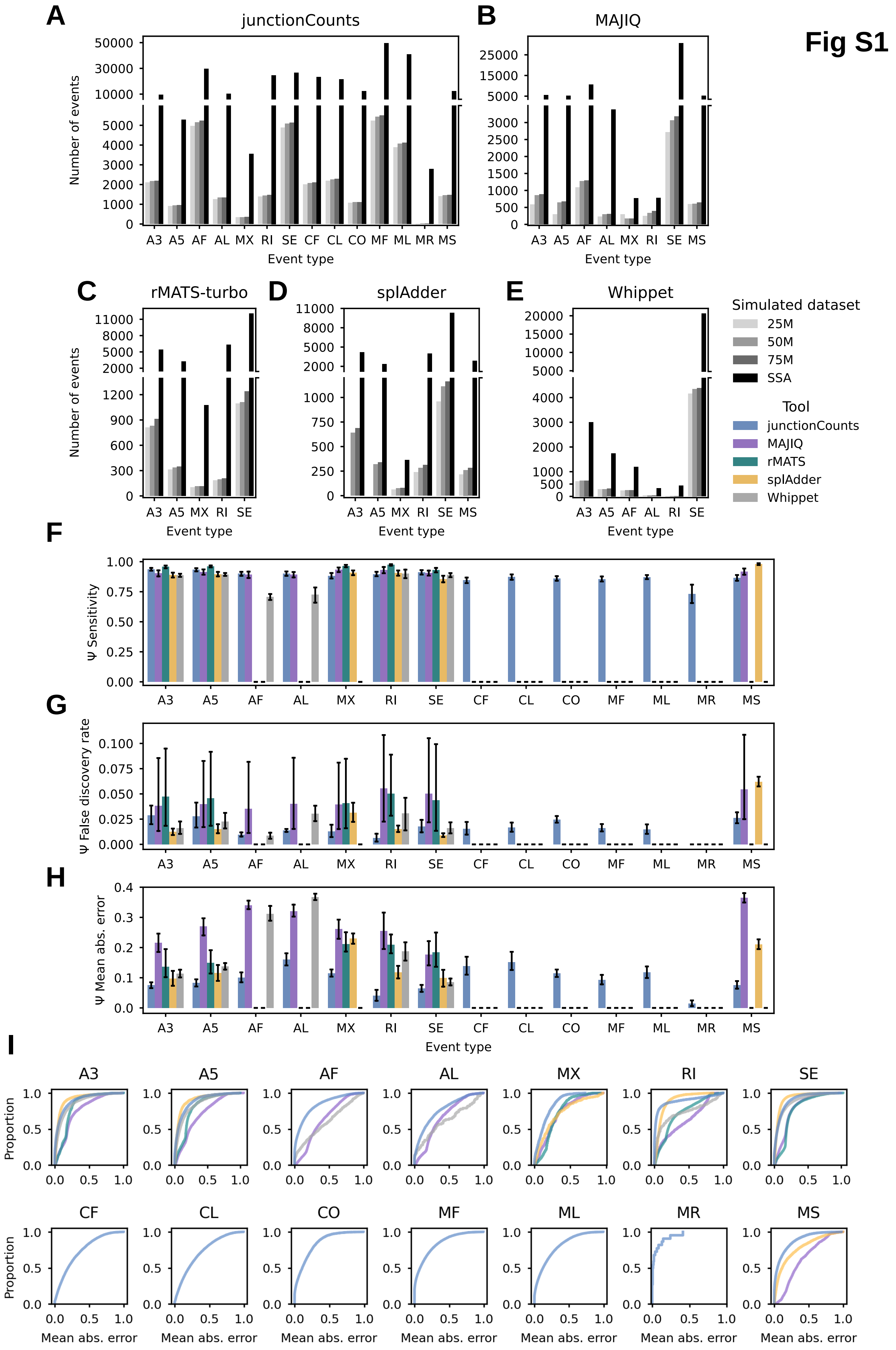
A junctionCounts
B MAJIQ
C rMATS-turbo
D splAdder
E Whippet
Number of events
Event type
A3 A5 AF AL MX RI SE CF CL CO MF ML MR MS
Simulated dataset
25M
50M
75M
SSA
Tool
junctionCounts
MAJIQ
rMATS
splAdder
Whippet
F
Ψ Sensitivity
G
Ψ False discovery rate
H
Ψ Mean abs. error
I
Proportion
Mean abs. error

Figure S1. Finer details of benchmarking experiment and results.
(A-E) The number of events, by event type, tested for each tool's performance on each simulated dataset. 25M, 50M and 75M refer to the library depths of the three mouse cerebellum and liver RNA-seq-based datasets. SSA refers to the dataset based on RNA-seq from spliceostatin A vs. DMSO treatment in human cells. (F) Sensitivity, or true positive rate, (G) false discovery rate and (H) mean absolute error of event detection by event type. (I) Empirical cumulative distribution function of mean absolute error at the PSI level by event type.

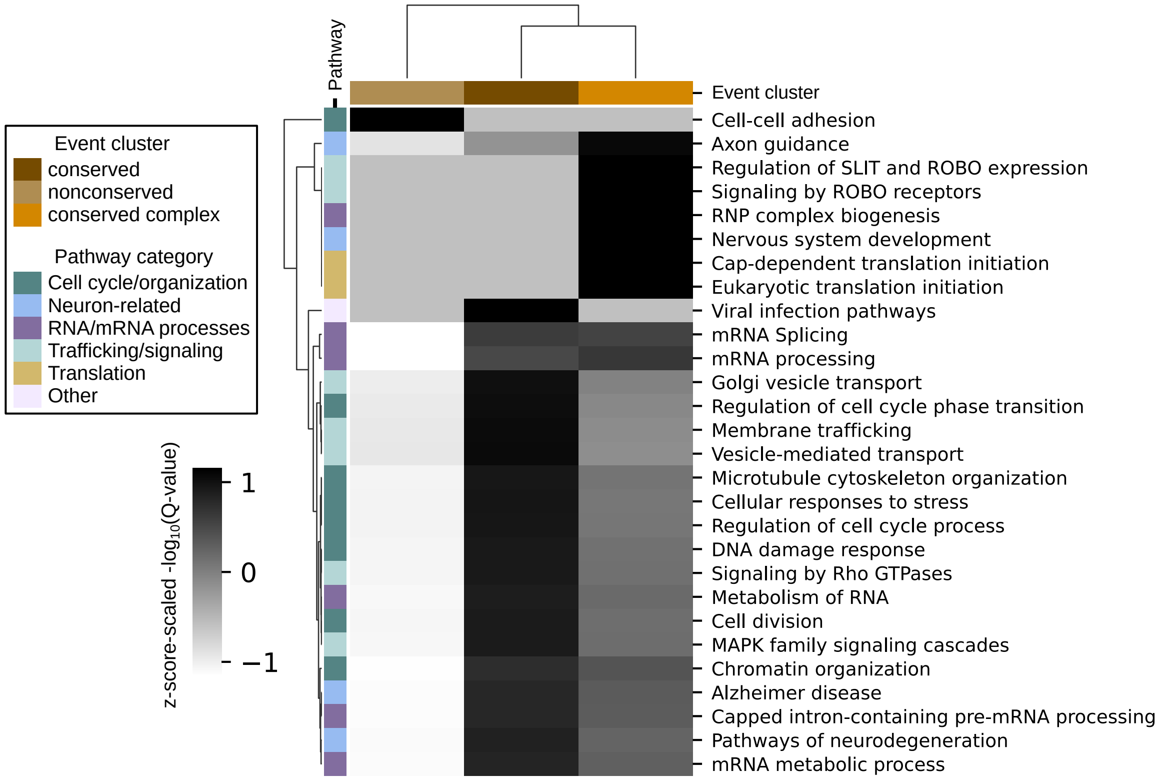

Figure S2. Metascape pathways for conserved and nonconserved event sets.
Heatmap of Z-score-scaled-log10(Q-value) of Metascape (38) pathway enrichment in event clusters. Conserved, conserved complex and nonconserved event clusters represent the set of genes with conserved splicing, the set of genes with conserved complex splicing events, and the set of genes with nonconserved splicing dynamics respectively.

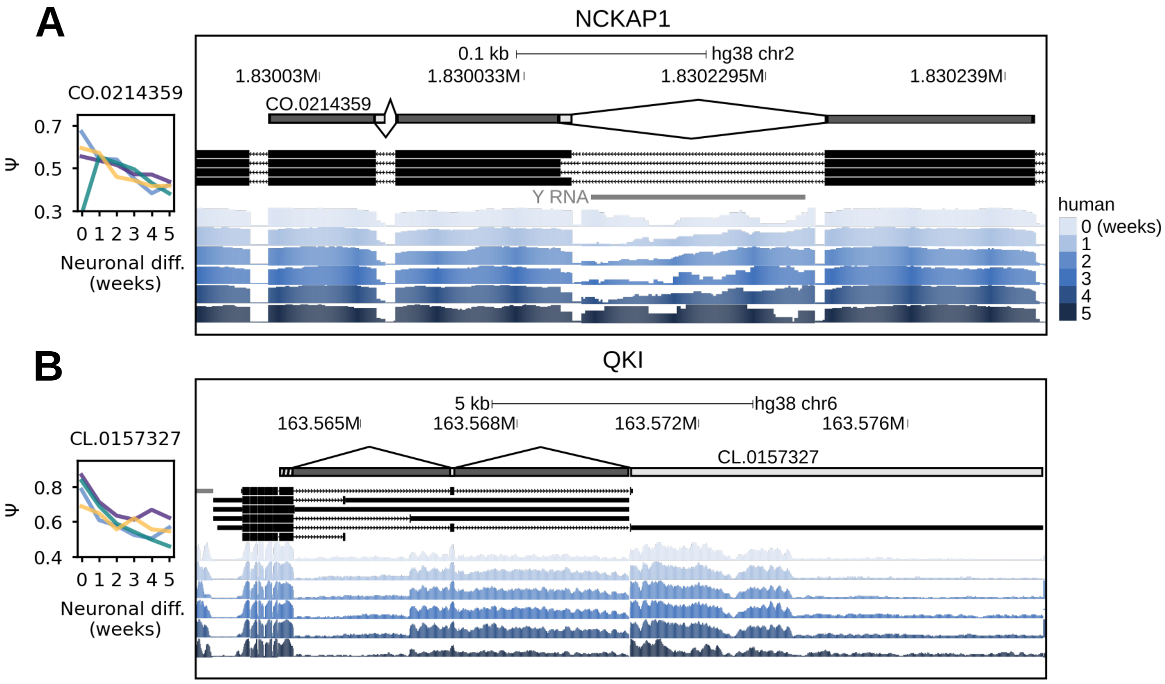

Figure S3. Examples of conserved complex events. UCSC Genome Browser snapshot of human read support at (A) a complex internal event in NCKAP1 and (B) a complex last exon event in QKI. The included form uses the splice junctions above the model and the excluded form uses those below it. PSI trajectory subplots to the left show the event's inclusion at each time point for chimpanzee, human, orangutan and macaque in purple, blue, green and yellow respectively.

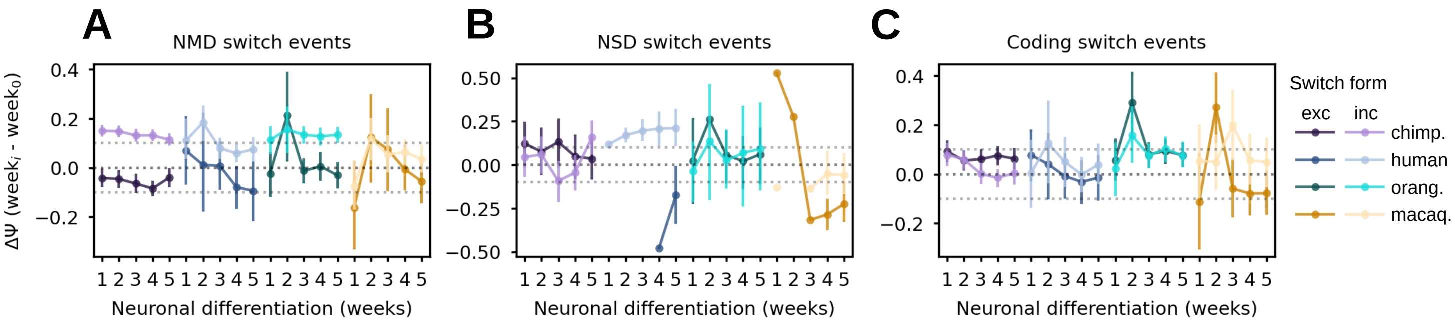

Figure S4. Directionality of switch forms in NMD, NSD and coding-to-noncoding switch events during primate neuronal differentiation. We expect that when the included form of a switch event confers a property (NMD/NSD/noncoding), its increased inclusion (dPSI) signals an increase in its abundance. If the excluded form confers the property, its decreased inclusion signals an increase in its abundance. (A-C) dPSI trajectories of NMD, NSD, coding-to-noncoding switch events respectively relative to week 0 over the time course. The switch form refers to the form confering the property: included (lighter color) or excluded (darker color).

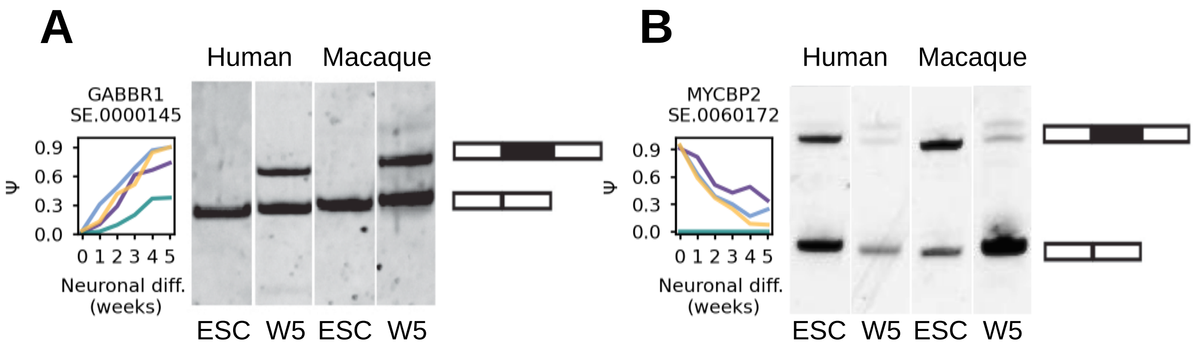

Figure S5. RT-PCR validation of two conserved SE events in human and macaque. (A) An SE event in GABBR1 which is completely excluded in ESC, but gradually included to a similar degree over the course of neuronal differentiation. (B) An SE event in MYCBP2 for which the included and excluded forms are similarly abundant in ESC, but by week 5 the excluded form becomes dominant. These RT-PCR data corroborate our findings by junctionCounts. PSI trajectory subplots to the left show the event's inclusion at each time point for chimpanzee, human, orangutan and macaque in purple, blue, green and yellow respectively.
